# The Association between Pro-Social Attitude and Reproductive Success Differs between Men and Women

**DOI:** 10.1371/journal.pone.0033489

**Published:** 2012-04-09

**Authors:** Martin Fieder, Susanne Huber

**Affiliations:** 1 Department of Anthropology, University of Vienna, Vienna, Austria; 2 Research Institute of Wildlife Ecology, University of Veterinary Medicine Vienna, Vienna, Austria; University of Turku, Finland

## Abstract

The evolution of pro-social attitude and cooperation in humans is under debate. Most of the knowledge on human cooperation results from laboratory experiments and theoretic modeling. Evolutionary explanations, however, rest upon fitness consequences. We therefore examined fitness correlates of pro-social behavior in a real life setting, analyzing data from the Wisconsin Longitudinal Study (n = 2545 men, 2967 women). We investigated whether pro-social attitude, proxied by self reported voluntary work, is associated with lifetime reproductive success. We find a sex difference in the association between pro-social attitude and offspring number. In men, a pro-social attitude was associated with higher offspring number, whereas in women, it was associated with lower offspring count. To our knowledge, this is the first study to demonstrate fitness consequences of pro-social behavior towards strangers. We conclude that analysing real life settings may help to explain the evolutionary forces leading to pro-social behavior in humans and speculate that these factors might differ between the sexes.

## Introduction

The nature and evolution of pro-social attitude and cooperation in humans is being intensively discussed. In particular, the selective mechanisms giving rise to pro-social behavior during human evolution are under debate. Proposed explanations involve both direct and indirect fitness benefits, multilevel selection models and various combinations of selection models [rev. 1]. Suggested mechanisms range amongst others from costly signaling, reputation building, and strong reciprocity, to altruistic punishment [1]–[6].

Most of our knowledge on human cooperation results from laboratory experiments and theoretical modeling [rev. 1]. Evolutionary explanations, however, rest upon fitness consequences. Yet, evidence for fitness correlates of pro-social behavior in modern humans is rare. Aim of the study was therefore to investigate fitness correlates of pro-social attitude in a real life setting, using data obtained from the Wisconsin Longitudinal Study. We examined whether pro-social attitude, proxied by self reported voluntary work, is associated with reproductive outcome. We further examined whether this association differed between men and women, because due to their differing reproductive potential, the evolutionary mechanisms leading to pro-social behavior might differ between the sexes.

## Methods

The Wisconsin Longitudinal Study is a long-term study of a random sample of 10,317 men and women who graduated from Wisconsin high schools in 1957, and were born in the years 1937–1940, which is thus only broadly representative of the whole American society. We used the surveys of 1990–1993 and 2003–2004 (telephone and mail surveys). As not all individuals participated in each survey, the final file contains a sample of 2545 men and 2967 women. We included the following variables in our analyses: i) number of biological children, obtained from survey in year 2004, when participants were 64 years of age and older and have thus typically finished reproduction; ii) sex of the participants encoded as 1 = male and 2 = female; iii) income (in 1000$) reported as the sum of wages before taxes during the previous 12 months, obtained from survey in year 1993 so that individuals were still participating in the work-force; iv) marital status, encoded as 1 = currently married, 2 = separated, 3 = divorced, 4 = widowed, 5 = never married; v) education, encoded in this sample of high school graduates as 1 = less than one year of college, 2 = 1 to 3 year college, 3 = bachelor degree, 4 = master degree and higher; and vi) as a proxy of pro-social attitude, whether or not the individual has performed voluntary work during the last ten years (self-reported: 1 = yes, 2 = no), obtained from survey in year 2004 (the only survey containing this variable).

We calculated and plotted separately for men and women, mean offspring number of individuals exhibiting and those not exhibiting a pro-social attitude. In addition, we initially performed a generalized linear mixed model of sex, marital status, education, voluntary work, and income as fixed factors and year of birth as random factor on the number of children on a Poisson error structure basis, which is appropriate for count data [7]–[8]. As including birth year as a random factor did not explain any variance, we eventually performed a generalized linear without this random factor. In addition, we limited this model to two-way interactions with voluntary work, which were reduced stepwise by the exclusion of non-significant interactions (see Supporting Information Tables S1, S2, S3). So our final generalized linear model was as follows: sex, marital status, education, income, voluntary work, and interaction between sex and voluntary work on number of children on the basis of a Poisson error structure.

## Results

**Figure 1:**
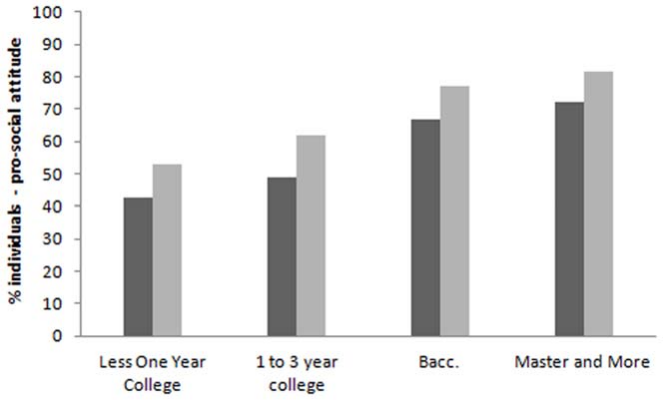
Percentage of men (black bars) and women (grey bars) exhibiting a pro-social attitude, separately for educational category.

In this sample of high school graduates, generally a higher percentage of women than men exhibited a pro-social attitude (women = 60.2%; men = 52.8%; χ2 = 29.9, P<0.0001). In both men and women, the percentage of individuals exhibiting a pro-social attitude increased with increasing education (Fig. 1).

**Figure 2:**
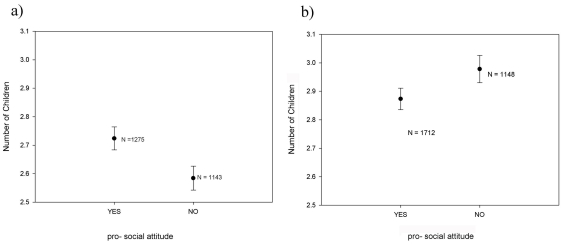
Offspring number (mean ± SE) of men exhibiting a pro-social attitude and men not exhibiting a pro-social attitude (a). Offspring number (mean ± SE) of women exhibiting a pro-social attitude and women not exhibiting a pro-social attitude (b).

In men, average offspring number was higher in individuals exhibiting than those not exhibiting a pro-social attitude (Fig. 2a). In contrast, in women, average offspring number was lower in individuals exhibiting than those not exhibiting a pro-social attitude (Fig. 2b). This sex difference in the association between voluntary work and offspring number is confirmed by a generalized linear model (Table 1) showing a significant positive effect of voluntary work and a significant negative effect of education and income on offspring number as well as a significant interaction between sex and voluntary work. In addition, it reveals that offspring number is significantly lower in never married and divorced than in married individuals, with no significant interaction between marital status and voluntary work (not shown).

**Table 1:** Generalized linear model of sex, voluntary work, marital status, education, and income on offspring number on the basis of a Poisson error structure.

Coefficients	Estimate	Std. Error	Z value	P
Intercept	1.1481	0.023	50.163	<0.001
Income	−0.0001	0.0002	−0.438	0.661
Voluntary work (reference: yes)	−0.0855	0.0267	−3.196	0.001
Education (reference: 1) 2	−0.0919	0.0248	−3.710	<0.001
3	−0.1373	0.0278	−4.937	<0.001
4	−0.2163	0.0293	−7.383	<0.001
Marital status (reference: 1) 2	−0.2093	0.2889	−0.725	0.469
3	−0.0874	0.0294	−2.970	0.003
4	−0.0052	0.0330	−0.158	0.874
5	−4.335	0.3777	−11.478	<0.001
Sex (reference: male)	0.0228	0.0249	0.913	0.361
Voluntary work:sex	0.0809	0.0356	2.274	0.023

Residual deviance: 3475.1 on 4711 df;

Education: 1 = less than one year of college, 2 = 1 to 3 year college, 3 = bachelor degree, 4 = master degree and higher; Marital status: 1 = currently married, 2 = separated, 3 = divorced, 4 = widowed, 5 = never married.

## Discussion

We find that in men, a pro-social attitude was associated with higher offspring number, representing a ‘real life’ confirmation of direct fitness effects of pro-social behavior, beyond mathematical modeling and lab experiments. Although few studies have demonstrated effects of altruistic behavior toward kin on reproductive success [e.g.], [ 9]–[12], the present results are novel as they demonstrate fitness consequences of pro-social behavior towards strangers. We assume costly signaling and reciprocity as potential underlying mechanisms for the positive association between pro-social attitude and offspring number found in men [13]–[16]. Studies from traditional societies, for instance, suggest that men engaged in costly activities may be attractive as mating partners [17]. Voluntary work has also been shown to increase a man's income [18], which in turn is associated with reproductive advantages in men [19]–[23].

In contrast, in women, exhibiting a pro-social attitude was associated with lower offspring count, which does not conform to evolutionary models based upon direct fitness benefits. Day and Devlin [18] likewise found that offspring number was usually higher in men but lower in women engaged in voluntary work. Yet, we found that more women than men exhibited a pro-social attitude. A possible explanation that pro-social women have fewer children because they do not marry but devote their lives to charitable work does not hold as we did not find any significant interaction between marital status and voluntary work. However, it should be noted that the data base does not include the information when marriage occurred in relation to voluntary work. We speculate that in women, a pro-social attitude might be rather associated with indirect benefits where women profit from altruism among kin. Findings of fitness enhancing effects of altruism toward kin among women support this argumentation [10], [12].

The study has limitations as our data base did not make it possible to get into more detail of the kind of voluntary work representing our proxy of pro-social attitude. Such details might be important in view of potential sex differences. In addition, our proxy was self reported voluntary work at age 54 years and older. So our results may also be interpreted that men having more offspring are more prone to do voluntary work than men with a smaller progeny, whereas the opposite is true for women, possibly because mother's and grandmother's duties do not leave enough time for voluntary work in women with more children. However, as pro-social attitude is most likely a matter of personality, we do not believe that such causality can be derived from our results.

We conclude that investigations beyond experimental situation in real life settings may help to resolve the discussion upon the evolutionary forces leading to pro-social behavior in humans. From our findings we speculate that owing to their high reproductive potential, direct fitness advantages might be more important in men, whereas in women, who are stronger limited than men in the overall number of offspring they can produce, maybe indirect fitness benefits might dominate.

## Supporting Information

Table S1 **Generalized linear model of sex, voluntary work, marital status, education, and income on offspring number on the basis of a Poisson error structure, including all two-way interactions with voluntary work.**
(DOC)

Table S2 **Generalized linear model of sex, voluntary work, marital status, education, and income on offspring number on the basis of a Poisson error structure, excluding the least significant interaction from Table S1.**
(DOC)

Table S3 **Generalized linear model of sex, voluntary work, marital status, education, and income on offspring number on the basis of a Poisson error structure, excluding the least significant interaction from Table S2.**
(DOC)
