# Supplementary material for: The Association between Pro-Social Attitude and Reproductive Success Differs between Men and Women
Source: PLoS One. 2012 Apr 9;7(4):e33489. doi: 10.1371/journal.pone.0033489 (PMC3322138; doi:10.1371/journal.pone.0033489)
Supplement: Table S1 — Generalized linear model of sex, voluntary work, marital status, education, and income on offspring number on the basis of a Poisson error structure, including all two-way interactions with voluntary work. (DOC) [file pone.0033489.s001.doc]

| **Coefficients** | **Estimate** | **Std. Error** | **Z value** | **P** |
| --- | --- | --- | --- | --- |
| Intercept | 1.142 | 0.0254 | 44.998 | <0.001 |
| Income | 0.00006 | 0.0003 | 0.021 | 0.983 |
| Voluntary work (reference: yes) | -0.0708 | 0.0376 | -1.884 | 0.060 |
| Education (reference: 1) 2 | -0.0815 | 0.0327 | -2.491 | 0.013 |
| 3 | -0.1447 | 0.0361 | -4.305 | <0.001 |
| 4 | -0.2178 | 0.0343 | -6.349 | <0.001 |
| Marital status (reference: 1) 2 | 0.1379 | 0.3784 | 0.364 | 0.716 |
| 3 | -0.1001 | 0.0410 | -2.441 | 0.015 |
| 4 | 0.0155 | 0.0449 | 0.344 | 0.731 |
| 5 | -5.091 | 0.7072 | -7.199 | <0.001 |
| Sex (reference: male) | 0.0254 | 0.0259 | 0.981 | 0.327 |
| Voluntary work:income | -0.0003 | 0.0005 | -0.618 | 0.537 |
| Voluntary work:education 2 | -0.0257 | 0.0502 | -0.512 | 0.608 |
| Voluntary work:education 3 | 0.0292 | 0.0603 | 0.484 | 0.629 |
| Voluntary work:education 4 | 0.0070 | 0.0669 | 0.105 | 0.917 |
| Voluntary work:marital status 2 | -0.6928 | 0.5861 | -1.182 | 0.237 |
| Voluntary work:marital status 3 | 0.0255 | 0.0590 | 0.433 | 0.665 |
| Voluntary work:marital status 4 | -0.0471 | 0.0664 | -0.710 | 0.478 |
| Voluntary work:marital status 5 | 1.354 | 0.8369 | 1.618 | 0.106 |
| Voluntary work:sex | 0.0748 | 0.0395 | 1.893 | 0.058 |

Residual deviance: 3469.0 on 4703 df;

Education: 1 = less than one year of college, 2 = 1 to 3 year college, 3 = bachelor degree, 4 = master degree and higher; Marital status: 1= currently married, 2 = separated, 3= divorced, 4= widowed, 5 = never married.
