# Supplementary material for: The Association between Pro-Social Attitude and Reproductive Success Differs between Men and Women
Source: PLoS One. 2012 Apr 9;7(4):e33489. doi: 10.1371/journal.pone.0033489 (PMC3322138; doi:10.1371/journal.pone.0033489)
Supplement: Table S2 — Generalized linear model of sex, voluntary work, marital status, education, and income on offspring number on the basis of a Poisson error structure, excluding the least significant interaction from Table S1. (DOC) [file pone.0033489.s002.doc]

| **Coefficients** | **Estimate** | **Std. Error** | **Z value** | **P** |
| --- | --- | --- | --- | --- |
| Intercept | 1.143 | 0.0247 | 46.280 | <0.001 |
| Income | -0.000009 | 0.0003 | -0.030 | 0.976 |
| Voluntary work (reference: yes) | -0.0723 | 0.0359 | -2.012 | 0.044 |
| Education (reference: 1) 2 | -0.0923 | 0.0248 | -3.720 | <0.001 |
| 3 | -0.1367 | 0.0278 | -4.912 | <0.001 |
| 4 | -0.2161 | 0.0293 | -7.373 | <0.001 |
| Marital status (reference: 1) 2 | -0.143 | 0.3783 | 0.378 | 0.705 |
| 3 | -0.0999 | 0.0410 | -2.436 | 0.015 |
| 4 | 0.0166 | 0.0449 | 0.369 | 0.712 |
| 5 | -5.092 | 0.7072 | -7.200 | <0.001 |
| Sex (reference: male) | 0.0253 | 0.0259 | 0.976 | 0.329 |
| Voluntary work:income | -0.0003 | 0.0005 | -0.571 | 0.568 |
| Voluntary work:marital status 2 | -0.702 | 0.5859 | -1.198 | 0.231 |
| Voluntary work:marital status 3 | 0.0248 | 0.0589 | 0.422 | 0.673 |
| Voluntary work:marital status 4 | -0.0468 | 0.0663 | -0.706 | 0.480 |
| Voluntary work:marital status 5 | 1.358 | 0.8369 | 1.622 | 0.105 |
| Voluntary work:sex | 0.0747 | 0.0395 | 1.893 | 0.058 |

Residual deviance: 3469.6 on 4706 df;

Education: 1 = less than one year of college, 2 = 1 to 3 year college, 3 = bachelor degree, 4 = master degree and higher; Marital status: 1= currently married, 2 = separated, 3= divorced, 4= widowed, 5 = never married.
