# Supplementary material for: The Association between Pro-Social Attitude and Reproductive Success Differs between Men and Women
Source: PLoS One. 2012 Apr 9;7(4):e33489. doi: 10.1371/journal.pone.0033489 (PMC3322138; doi:10.1371/journal.pone.0033489)
Supplement: Table S3 — Generalized linear model of sex, voluntary work, marital status, education, and income on offspring number on the basis of a Poisson error structure, excluding the least significant interaction from Table S2. (DOC) [file pone.0033489.s003.doc]

| **Coefficients** | **Estimate** | **Std. Error** | **Z value** | **P** |
| --- | --- | --- | --- | --- |
| Intercept | 1.1482 | 0.023 | 50.009 | <0.001 |
| Income | -0.0001 | 0.0002 | -0.030 | 0.976 |
| Voluntary work (reference: yes) | -0.0857 | 0.0273 | -3.145 | 0.002 |
| Education (reference: 1) 2 | -0.0927 | 0.0248 | -3.741 | <0.001 |
| 3 | -0.1365 | 0.0278 | -4.907 | <0.001 |
| 4 | -0.2160 | 0.0293 | -7.371 | <0.001 |
| Marital status (reference: 1) 2 | 0.1431 | 0.3783 | 0.378 | 0.705 |
| 3 | -0.0995 | 0.0410 | -2.426 | 0.015 |
| 4 | 0.0165 | 0.0449 | 0.366 | 0.714 |
| 5 | -5.0915 | 0.7072 | -7.199 | <0.001 |
| Sex (reference: male) | 0.0216 | 0.0251 | 0.861 | 0.389 |
| Voluntary work:marital status 2 | -0.6999 | 0.5859 | -1.195 | 0.232 |
| Voluntary work:marital status 3 | 0.0242 | 0.0589 | 0.411 | 0.681 |
| Voluntary work:marital status 4 | -0.0463 | 0.0663 | -0.698 | 0.485 |
| Voluntary work:marital status 5 | 1.3576 | 0.8369 | 1.622 | 0.105 |
| Voluntary work:sex | 0.0837 | 0.0362 | 2.309 | 0.021 |

Residual deviance: 3469.9 on 4707 df;

Education: 1 = less than one year of college, 2 = 1 to 3 year college, 3 = bachelor degree, 4 = master degree and higher; Marital status: 1= currently married, 2 = separated, 3= divorced, 4= widowed, 5 = never married.
